# Discrepancy Between Preoperative and Intraoperative Central Corneal Epithelial Thickness During Alcohol‐Assisted PRK

**DOI:** 10.1155/joph/4117242

**Published:** 2026-10-11

**Authors:** Dana Barequet, Nadav Levinger, Oren Golan, Samuel Levinger, Irina Barequet

**Affiliations:** ^1^ Division of Ophthalmology Tel Aviv Medical Center Tel Aviv Israel tau.ac.il; ^2^ Enaim Refractive Surgery Center Tel Aviv Israel; ^3^ Goldschleger Eye Institute Sheba Medical Center Tel Hashomer Israel sheba.co.il

## Abstract

**Purpose:**

To evaluate the agreement between preoperative optical coherence tomography (OCT)–derived central corneal epithelial thickness (CET) and an intraoperative epithelial thickness estimate derived from optical coherence pachymetry (OCP) during alcohol‐assisted photorefractive keratectomy (PRK).

**Methods:**

A retrospective analysis was performed on 116 eyes of 58 consecutive patients who underwent bilateral myopic alcohol‐assisted PRK between June 2020 and October 2021. Preoperative CET was assessed using anterior‐segment OCT. The OCP‐derived intraoperative epithelial thickness estimate was calculated as the difference between total corneal pachymetry readings obtained immediately before and after epithelial removal. Demographic, preoperative clinical, and refractive data, as well as intraoperative laser parameters, were collected.

**Results:**

Mean age was 26.31 ± 7.44 years (range, 18–45), and 63.8% of patients were female. Mean sphere and spherical equivalent were −3.49 ± 1.63D and −3.16 ± 1.63D, respectively. Mean preoperative OCT‐derived CET was 53.2 ± 3.5  μm (range, 42–69  μm), whereas the mean OCP‐derived intraoperative CET was significantly higher at 59.8 ± 14.8  μm (range, 21–99  μm; *p* < 0.0001). This discrepancy was consistent in both eyes of each patient. The intraoperative estimate exceeded the preoperative OCT‐derived CET in 73.3% of eyes. Bland–Altman analysis demonstrated poor agreement between the two approaches, with wide limits of agreement from −15.7  μm to +34.8  μm. Preoperatively, 98.3% of eyes had CET values within the 40–60‐μm range, compared with 44.8% of the OCP‐derived intraoperative estimates. The difference between measurements significantly correlated with preoperative corneal thickness (*r* = 0.45, *p* < 0.001) and intraoperative CET (*r* = 0.97, *p* < 0.0001) but not refractive error or intraoperative and environmental parameters.

**Conclusion:**

Preoperative OCT‐derived CET and OCP‐derived intraoperative CET demonstrate a systematic discrepancy and poor agreement during alcohol‐assisted PRK. The intraoperative estimates were higher on average and substantially more variable than preoperative OCT measurements. These differences may have implications for surface ablation procedures, particularly transepithelial PRK, in which an assumed epithelial thickness profile is incorporated into treatment planning.

## 1. Introduction

The corneal epithelium has a distinct anatomical and optical role [1], contributing approximately 1.0 diopter to the cornea’s refractive power [2]. Its average thickness typically ranges from 50 to 60 µm [1, 3] but may vary with age [4, 5], gender [4, 6], ethnicity, and refractive errors [6, 7]. Changes in central epithelial thickness (CET) have also been associated with pathologic conditions, such as contact lens (CL) wear [8], dry eye disease [9, 10], and keratoconus [11].

Modern imaging modalities, including very high frequency (VHF) ultrasound [3], confocal microscopy [12, 13], and optical coherence tomography (OCT) [10, 14, 15], allow precise, noninvasive mapping of the epithelial layer. These measurements assist in diagnosing and managing corneal diseases and serve as an important tool in preoperative refractive surgery evaluation.

Optical coherence pachymetry system (OCP, Heidelberg Engineering, Lübeck, Germany), integrated within the SCHWIND Amaris excimer laser, enables continuous, noncontact, intraoperative measurements of the central corneal thickness during refractive procedures. Its capability to intraoperatively track corneal thickness has demonstrated high reproducibility of intraoperative corneal changes, such as the flap thickness and the residual stromal thickness [16–19].

In photorefractive keratectomy (PRK), the epithelium is removed entirely before stromal ablation, eliminating its direct effect on the refractive outcome. Recently, transepithelial PRK (t‐PRK) has emerged as a popular alternative, which applies a standardized ablation profile that incorporates the epithelium (typically 55 μm centrally and 65 μm peripherally) followed by stromal ablation. Therefore, individual variability and deviation from this assumed profile may lead to incomplete epithelial removal or excessive treatment, potentially influencing refractive accuracy.

This study aimed to compare preoperative OCT‐derived CET with an OCP‐derived intraoperative epithelial thickness estimate during alcohol‐assisted myopic PRK and to characterize the magnitude and variability of the discrepancy between these approaches. The primary endpoint was the difference between these two measurements.

## 2. Materials and Methods

### 2.1. Patient and Study Design

This retrospective analysis included consecutive patients who underwent bilateral, simultaneous, alcohol‐assisted myopic PRK between June 2020 and October 2021. Inclusion criteria were a minimum corneal thickness of 480 µm, the absence of contraindications for laser refractive surgery, and refractive errors suitable for aspheric PRK. Eyes with complete preoperative and intraoperative data, as outlined below, were included; unilateral cases or eyes which had missing data were excluded.

Written informed consent was obtained from each participant prior to the surgery. The study adhered to the tenets of the Declaration of Helsinki and was approved by the Institutional Review Board. As this was a retrospective consecutive series, the sample size was determined by the number of eligible cases with complete data during the predefined study period.

### 2.2. Preoperative Examination

A detailed ophthalmic and systemic history was obtained for all patients. Contact lens wear was discontinued for at least 10 days prior to evaluation and surgery, depending on the type of lenses worn. Preoperative assessment included uncorrected and best‐corrected distance visual acuity, as well as manifest and cycloplegic refraction. A full ophthalmic examination was performed, encompassing slit‐lamp examination, intraocular pressure measurement (Goldmann applanation tonometry), corneal surface evaluation with fluorescein staining and tear breakup time, and dilated fundus examination. Additionally, all patients underwent MS‐39 anterior‐segment OCT/Placido system (CSO, Florence, Italy) which provided epithelial mapping and corneal thickness measurements.

### 2.3. Surgical Technique

All surgeries were performed using a uniform technique by two experienced refractive surgeons (I.S.B. and O.G.) utilizing the SCHWIND Amaris 500E excimer laser platform (SCHWIND eye‐tech‐solutions GmbH, Kleinostheim, Germany). The system’s integrated OCP module provided intraoperative central pachymetry, and the ablation profile was calculated using ORK‐CAM software, targeting emmetropia in all cases.

In the preparation area, each eye received one drop of topical 0.4% oxybuprocaine hydrochloride and moxifloxacin 0.5% (Vigamox, Alcon Laboratories, Inc., Fort Worth, TX). After lid cleaning with 5% povidone‐iodine solution, patients were positioned supine under the laser system in the operating room. A sterile drape and lid speculum were applied.

An initial OCP measurement of central corneal thickness was obtained after positioning and alignment of the eye under the excimer laser and before epithelial removal. An additional drop of oxybuprocaine was then instilled, and epithelial delamination was performed using an 8.5‐mm trephine well filled with 20% ethanol for 30 s. After removal of the alcohol and complete mechanical epithelial debridement, a second central OCP measurement was obtained before stromal laser ablation, using the same laser platform and central alignment.

Laser ablation was performed according to the planned profile. Mitomycin‐C 0.02% was applied for 10–30 s, depending on ablation depth, followed by irrigation with chilled balanced salt solution. A bandage contact lens was placed and removed 4–6 days later.

Postoperative treatment included topical moxifloxacin 0.5% four times daily for 1 week, loteprednol 0.5% (Lotemax, Bausch & Lomb, Inc.) four times daily for one month (then tapered over the next month), and preservative‐free artificial tears as needed.

### 2.4. Data Collection

Collected variables included demographic information, refractive error, preoperative corneal and epithelial thickness, and intraoperative parameters such as treatment and ablation times, ablation zone diameter, cyclotorsion compensation, room humidity and temperature, and planned ablation depth. Three OCP readings of total central corneal thickness were recorded: (1) after initial alignment and before epithelial removal, (2) after complete epithelial removal and before stromal ablation, and (3) after completion of stromal ablation. The OCP‐derived intraoperative epithelial thickness estimate was calculated as the difference between the first and second pachymetry measurements. Because OCP measures total corneal thickness rather than the epithelial layer directly, this calculated value represents an indirect estimate of epithelial thickness. Repeated measurements at the same surgical time point and formal OCP quality‐control metrics were not available for this retrospective analysis.

### 2.5. Statistical Analysis

Data were recorded using Microsoft Excel 2010 and analyzed with SPSS Version 25 (IBM Corp., Chicago, IL, USA). Continuous variables, such as epithelial thickness, were compared between groups based on dichotomous demographic data using the independent sample *t*‐test. Effect size for the primary comparison was expressed as paired‐samples Cohen’s dz, calculated as the mean within‐eye difference divided by the standard deviation of the paired differences. To avoid the influence of intereye dependence, the effect size was calculated using the first‐operated‐eye analysis. For paired variables, comparisons were performed using the paired‐samples *t*‐test. Correlations between continuous variables were assessed using Pearson’s correlation coefficient. Correlation analyses were considered exploratory, and no adjustment for multiple comparisons was applied. As both eyes of each patient were included, the primary comparison between preoperative OCT‐derived CET and the OCP‐derived intraoperative epithelial thickness estimate was also performed separately for the first and second operated eyes to assess whether the observed difference persisted when only one eye per patient was included in each analysis. All statistical tests were two‐tailed, and a *p* value < 0.05 was considered statistically significant.

## 3. Results

A total of 116 eyes of 58 consecutive patients were included in the analysis. The mean age was 26.31 ± 7.44 years (range, 18–45), and 63.8% of participants were females. The mean preoperative spherical power was −3.49 ± 1.63 D (range, −8.00 to −0.75 D), the mean cylinder was −0.66 ± 0.51 D (range, −2.75 to 0 D), and the mean spherical equivalent was −3.16 ± 1.63 D (range, −8.175 to −0.25 D). Baseline demographic and refractive data are summarized in Table 1.

**TABLE 1:** Associations of preoperative CET, OCP‐derived intraoperative CET estimate, and thickness discrepancy with demographic and preoperative variables.

	Patients *n* = 58	Eyes *n* = 116	Preoperative CET		Intraoperative CET		Deviation in thickness	
			*p*	*r*_*p*_	*p*	*r*_*p*_	*p*	*r*_*p*_
Age, years (mean ± SD)	26.31 ± 7.44		**0.005**	0.26	0.47	0.07	0.95	0.01
Gender‐female, *n* (%)	37 (63.8)		0.91		0.11		0.08	
Refractive errors, D (mean ± SD)								
Sphere		−3.49 ± 1.63	0.31	0.10	0.33	0.09	0.44	0.07
Cylinder		−0.66 ± 0.51	0.15	−0.14	0.75	−0.03	0.99	0.002
Spherical equivalent		−3.16 ± 1.63	0.21	0.12	0.31	0.10	0.44	0.07
Preoperative central corneal thickness, µm (mean ± SD)		538.94 ± 31.26	**0.03**	0.20	**< 0.0001**	0.47	**< 0.0001**	0.45
Preoperative CET, µm (mean ± SD)		53.22 ± 3.47			**< 0.0001**	0.38	0.11	0.15
Intraoperative CET, µm (mean ± SD)		59.78 ± 14.80	**< 0.0001**	0.38			**< 0.0001**	0.97
Deviation in thickness, µm (mean ± SD)		6.56 ± 13.82	0.11	0.15	**< 0.0001**	0.97		

*Note:* The bold values indicate statistically significance.

Abbreviation: CET, central epithelial thickness.

The mean preoperative OCT‐derived CET was 53.22 ± 3.47 µm (range, 42–69 µm). The mean OCP‐derived intraoperative CET was significantly higher at 59.78 ± 14.80 µm (range, 21–99 µm; *p* < 0.0001). The OCP‐derived intraoperative estimate exceeded the preoperative OCT‐derived CET in 73.3% of eyes (Figure 1). To account for the potential dependence of bilateral observations, the comparison was additionally performed separately for the first and second operated eyes, thereby including one eye per patient in each analysis. The difference remained statistically significant in both the first operated eyes (*p* < 0.0001) and the second operated eyes (*p* < 0.01). *n* was the first‐operated‐eye analysis, and the mean difference was 7.29 ± 13.2 µm, corresponding to a paired‐samples Cohen’s dz of 0.55. The mean intereye difference in deviation was not significant (7.29 ± 13.2 µm vs. 5.83 ± 14.4 µm; *p* = 0.59).

**FIGURE 1:**
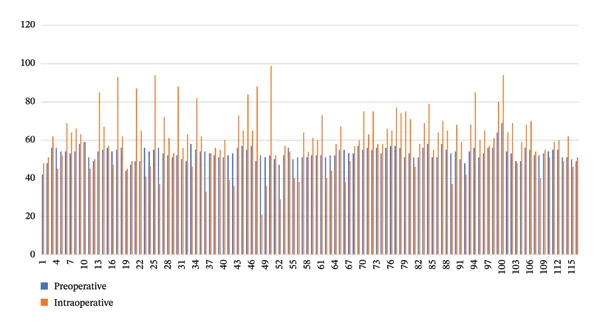
Preoperative OCT‐derived central epithelial thickness and OCP‐derived intraoperative epithelial thickness estimates for each operated eye.

Bland–Altman analysis demonstrated poor agreement between preoperative OCT‐derived CET and the OCP‐derived intraoperative epithelial thickness estimate, with wide limits of agreement ranging from −15.7 µm to +34.8 µm, indicating that the two approaches should not be considered interchangeable (Figure 2).

**FIGURE 2:**
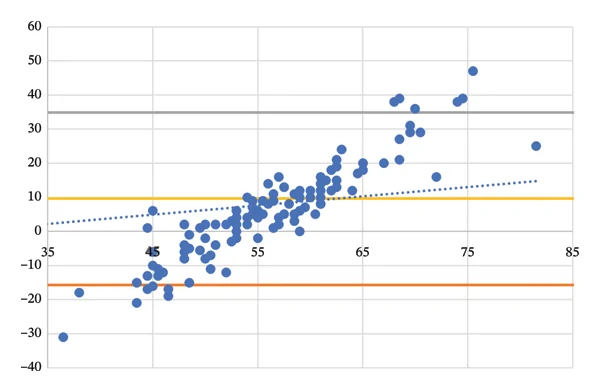
Bland–Altman plot comparing preoperative OCT‐derived central epithelial thickness and the OCP‐derived intraoperative epithelial thickness estimate. Poor agreement was observed between the two approaches, with wide limits of agreement ranging from −15.7 to +34.8 µm, indicating that the measurements should not be considered interchangeable.

Preoperatively, 98.3% of eyes (114/116) had CET values within the 40–60 µm range; only two eyes (1.7%) exceeded this range (64 µm and 69 µm). Intraoperatively, however, only 44.8% of eyes (52/116) had OCP‐derived estimates within this range, whereas 8.6% (10 eyes) were below and 46.6% (54 eyes) were above this range (Figure 3).

**FIGURE 3:**
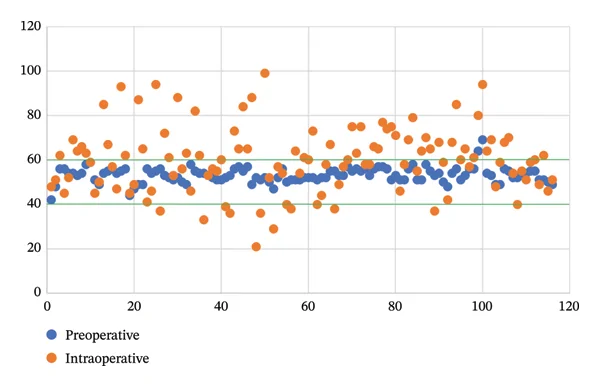
Distribution of preoperative OCT‐derived central epithelial thickness and OCP‐derived intraoperative epithelial thickness estimates. Preoperatively, 98.3% of eyes had CET values within the 40–60‐µm range, compared with 44.8% of the intraoperative estimates.

Correlation analysis demonstrated that preoperative CET was significantly correlated with age (*r* = 0.26, *p* = 0.005) and preoperative central corneal thickness (*r* = 0.20, *p* = 0.03). The OCP‐derived intraoperative epithelial thickness estimate was significantly correlated with preoperative central corneal thickness (*r* = 0.47, *p* < 0.0001) and preoperative CET (*r* = 0.38, *p* < 0.0001). The deviation between preoperative and intraoperative measurements was significantly correlated with preoperative central corneal thickness (*r* = 0.45, *p* < 0.0001) and, most strongly, with the OCP‐derived intraoperative epithelial thickness estimate (*r* = 0.97, *p* < 0.0001). No significant correlations were identified between the thickness deviation and age, refractive error, or the assessed intraoperative and environmental parameters.

## 4. Discussion

This study demonstrated a systematic discrepancy between preoperative OCT‐derived CET and an intraoperative epithelial thickness estimate derived from serial OCP pachymetry during alcohol‐assisted PRK. The OCP‐derived estimates were higher on average and demonstrated substantially greater variability than preoperative OCT measurements.

Various methods for assessing epithelial thickness have been described in the literature, including VHF digital ultrasound [1, 3], optical pachymetry [7, 20], anterior‐segment OCT [4–6, 8–10, 14, 15, 21], and confocal microscopy [12, 13, 22]. These tools are limited to the preoperative evaluation of refractive surgery candidates. Although these modalities provide valuable preoperative structural information, they may not accurately reflect real‐time intraoperative epithelial changes which could influence ablation precision.

Our mean preoperative CET of 53.2 ± 3.5 µm is comparable to those previously reported for healthy corneas, which typically range between 48 ± 5 [20] and 59.9 ± 5.9 μm [7]. In contrast to the relatively narrow distribution of preoperative OCT‐derived CET, the OCP‐derived intraoperative estimates demonstrated substantially greater variability, ranging from 21 to 99 µm. Values at the extremes of this range are outside those typically reported for physiological epithelial thickness and therefore warrant cautious interpretation. Because the intraoperative estimate is calculated from the difference between two total corneal pachymetry measurements, variability in either measurement is propagated into the calculated value. The observed distribution may therefore reflect a combination of true intraoperative tissue changes and measurement‐related variability.

Notably, 98.3% of eyes in our study had a preoperative CET within the 40–60 µm range commonly reported in the literature. In contrast, only 44.8% of eyes had intraoperative CET within this range, with the remainder exhibiting either thinner or thicker epithelium. Several factors may contribute to the observed discrepancy. While measurement inaccuracy could be one explanation, previous studies have validated the reliability and reproducibility of the OCP system compared to other modalities. The difference between pre‐ and intraoperative CET may be attributed to real‐time physiological changes during surgery, including epithelial swelling from topical anesthetics and corneal dehydration during laser ablation [23].

Topical anesthetic agents are known to affect epithelial and endothelial pump activity, leading to transient corneal swelling [24, 25]. Mukhopadhyay et al. [24] reported considerable interindividual variability in swelling responses, ranging from more than a 10‐µm decrease to over a 30‐µm increase in individual cases. Weekers et al. [25] suggested that anesthetic agents alter the Na+/K + ATPase pump function in the corneal endothelium, increasing osmotic pressure and promoting epithelial and stromal hydration. Variations in environmental humidity and temperature during laser ablation may further modify epithelial hydration status. Although our study found no direct correlation between CET deviation and these intraoperative parameters, it remains plausible that subtle, patient‐specific hydration differences contribute to the observed variability.

The potential clinical relevance of these findings may be greatest in t‐PRK, in which the treatment algorithm incorporates an assumed epithelial thickness profile, typically approximately 55 µm centrally and 65 µm peripherally [26–28]. Previous theoretical and clinical studies have suggested that deviations from the assumed epithelial profile may influence the amount of stromal ablation and postoperative refractive outcome [29–31]. For example, Jun et al. [31] reported that thinner epithelia (< 50 µm) were associated with a hyperopic shift postoperatively, whereas thicker epithelia (≥ 60 µm) produced a myopic shift. These findings provide a rationale for further investigation of epithelial thickness variability in surface ablation procedures. However, the present study did not evaluate postoperative refractive outcomes, enhancement rates, or correlations between the observed thickness discrepancy and postoperative refractive error.

Limitations of this study include its retrospective design, which may be subject to selection bias, although data from consecutive eyes were collected to mitigate this. The sample size was determined by the number of eligible cases available during the predefined study period, and no a priori sample size calculation was performed. An important limitation is that preoperative and intraoperative epithelial thickness were assessed using fundamentally different approaches. Preoperative OCT provides a direct measurement of the epithelial layer, whereas the intraoperative estimate was derived indirectly from the difference between two measurements of total corneal thickness. Therefore, the observed discrepancy may reflect not only physiological epithelial changes but also stromal hydration changes, alignment variability, topical anesthetic effects, epithelial irregularity, and measurement noise. The present study cannot determine the relative contribution of these factors. A further limitation is that repeated OCP measurements at each surgical time point were not available, precluding formal assessment of intrasession repeatability or reproducibility. Because the intraoperative epithelial thickness estimate represents the difference between two pachymetry measurements, measurement variability at either time point may be amplified in the calculated value and may partly explain the wide observed range. In addition, both eyes of each patient were included in the overall analysis, and analyses based on the complete cohort did not explicitly account for within‐subject intereye correlation, potentially resulting in underestimated standard errors and smaller *p* values. However, the primary comparison was also performed separately for the first and second operated eyes, thereby including only one eye per patient in each analysis, and the observed difference remained statistically significant in both groups. The correlation analyses were exploratory and were not adjusted for multiple comparisons. Finally, postoperative refractive outcomes and enhancement rates were not evaluated; therefore, the present study cannot determine whether the observed measurement discrepancy has a clinically meaningful effect on refractive outcomes. Taken together, the consistent direction of the discrepancy across fellow eyes supports the robustness of the primary observation of intraoperative changes.

## 5. Conclusion

This study identified a systematic discrepancy and poor agreement between preoperative OCT‐derived central epithelial thickness and an OCP‐derived intraoperative epithelial thickness estimate during alcohol‐assisted PRK. The intraoperative estimates were higher on average and demonstrated substantially greater variability than preoperative OCT measurements, indicating that the two approaches should not be considered interchangeable. These findings suggest that intraoperative differences in epithelial thickness estimation may have implications for surface ablation procedures, particularly t‐PRK, in which an assumed epithelial thickness profile is incorporated into the treatment algorithm. Further prospective studies correlating these findings with postoperative refractive outcomes may help clarify their clinical significance.

## Funding

The authors have nothing to report.

## Conflicts of Interest

The authors declare no conflicts of interest.

## Data Availability

The datasets used and/or analyzed during the current study are available from the corresponding author on reasonable request.
